# Metals in Cow Milk and Soy Beverages: Is There a Concern?

**DOI:** 10.3390/toxics11121013

**Published:** 2023-12-11

**Authors:** Vanda Lopes Andrade, Iolanda Ribeiro, A. P. Marreilha dos Santos, Michael Aschner, Maria Luisa Mateus

**Affiliations:** 1 Research Institute for Medicines (iMed.ULisboa), Faculty of Pharmacy, Universidade de Lisboa, 1649-003 Lisboa, Portugal; vanda.andrade@esa.ipsantarem.pt (V.L.A.); santos-ana1@campus.ul.pt (A.P.M.d.S.); 2 Life Quality Research Centre (CIEQV), IPSantarem/IPLeiria, 2040-413 Rio Maior, Portugal; 3 Faculty of Sciences, Universidade de Lisboa, Campo Grande, 1749-016 Lisboa, Portugal; 4 Department of Molecular Pharmacology, Albert Einstein College of Medicine, Forchheimer Building, Room 209, Bronx, NY 10461, USA; michael.aschner@einsteinmed.edu

**Keywords:** metals, cow’s milk, soy beverage, atomic absorption spectrometry

## Abstract

Nowadays, there is an increased consumption of plant-based protein beverages like soy beverages (SBs) as substitutes for cow milk (CM). Both accumulate toxic metals like lead (Pb), cadmium (Cd), and manganese (Mn), which, although essential, are neurotoxic at high levels. Metals can also perturb the normal development of children. This work aimed to evaluate these metal concentrations in CM and SB purchased on the Portuguese market. After validation of the method, linearity of calibration curves, work range, detection and quantification limits, and selectivity, metals were determined in 14 CM and 14 SB brands using atomic absorption spectrometry. The values were compared between CM and SB and with permissible limit values. Soy beverages had significantly (*p* < 0.05) higher concentrations of Cd (5.6 ± 4.2 µg/L) and Mn (117.4 ± 30.3) µg/L) than CM (2.15 ± 1.84 µg/L and 5.93 ± 1.21 µg/L, respectively); the Pb concentrations in CM (19.3 ± 12.1 µg/L) were not significantly (*p* > 0.05) higher than in SB (13.4 ± 9.6 µg/L). These values were similar to other studies and close to but under permissible limit values. Nevertheless, due to the toxicity and bioaccumulation of metals, the fact that these foods are routinely ingested by all ages, mainly children, and represent key ingredients in many processed foods, including baby foods, we suggest strict surveying of metal levels in CM and SBs.

## 1. Introduction

Milk and its derivatives constitute one of the fundamental components of the human diet, particularly due to their protein and mineral content [1].

Milk is a complex mixture of bioactive substances that helps to promote the growth and development of infants [2]. It is widely consumed by children and adults and, consequently, is a very important food item [3].

The mineral content of cow’s milk (CM) is influenced by various factors, including a cow’s genetics, stage of lactation, milk solid composition, and intake and bioavailability of minerals in the diet [4,5,6,7].

In the last few decades, the increased consumption of plant-based protein as a substitution for animal sources has been seen globally [8,9]. Soy beverages (SBs) are plant-based protein-rich beverages that are produced from soybeans; they are a steady emulsion of water, oil, and protein. Soybean protein has a high nutritional quality, but there are concerns regarding its lipid content, as soy oil is rich in polyunsaturated fatty acids. Potassium, calcium, and magnesium are among the main minerals, with phosphorus and calcium being present at concentrations up to 20% higher than those present in cow milk-based infant formulas. However, SBs have lower energetic levels and contain fewer carbohydrates, fats, calcium, and vitamin B12 than CM. Along with similar vegetable-based milk, SB can be utilized as an alternative to dairy milk by individuals who are vegan, lactose intolerant, or allergic to CM, which is one of the most common food allergies in infancy. It can also be a substitute for CM where CM is not accessible in adequate amounts [10,11,12,13,14]. Along with options such as milk-hydrolyzed formulas for allergic subjects and lactose-free milk for lactose intolerant subjects, CM and SBs are nowadays the two main nutritive beverages ingested by humans at birth, during childhood, and for a lifetime, and they are also important ingredients in many processed foods including baby foods [15,16,17,18].

Although milk is a perfect source of many active biomolecules important for people’s health, it may contain chemical hazards such as heavy metals [4,6,7,9,19]. In fact, both SBs [20,21,22,23] and CM [24,25,26,27] may become contaminated with heavy metals like lead (Pb), cadmium (Cd), and manganese (Mn). Heavy metals are introduced into the environment by natural and anthropogenic factors. Metal mining, smelters, trash dumping, and incineration are some of the main sources of heavy metals produced by humans. Furthermore, the high use of chemical fertilizers and pesticides has increased the accumulation of heavy metals in soils and plants. The contamination and bioaccumulation of these contaminants in water sources led to the introduction of metals into food such as vegetables, milk, meat, fish, fruit juices, etc. The ingestion of these contaminated foods can cause damage to human health. Concerning the food products discussed in this study, the contamination that can occur during their manufacture should not be ignored [28,29].

In this study, these three metals were evaluated given their importance from the toxicological point of view, including their accumulation in the human body and their deleterious effects during the development of children [30].

Lead causes central nervous system disorders and anemia, damages the kidneys, liver, heart, blood vessels, immune system, genital system, and digestive tract. Furthermore, lead may trigger the development of various cancers [31,32]. Chronic Cd exposure is known to induce chronic renal tubular disease and may also cause hypertension and harm the cardiovascular and skeletal systems. Neurologic disorders can also occur. There are also several studies that have examined the relationship between the oral intake of Cd and cancer in humans [33,34].

Despite being an essential mineral, excessive exposure to manganese increases the risk of adverse neurological effects, especially when exposure occurs during childhood [35]. This metal can be found at high levels in plants such as soy and rice, which may constitute a matter of concern when well-meaning but inadequately informed parents perceive plant-based beverages such as soy or rice beverages as an alternative to infant formula [21,36,37].

Thus, according to a report from the Subcommittee on Economic and Consumer Policy of the Committee on Oversight and Reform, U.S. House of Representatives [15], there is a great concern regarding Pb and Cd exposure from baby food. CM and SBs alone or as ingredients of many processed foods can contribute to this heavy metal exposure [9,16,38]. Even so, as reported by another Subcommittee Report [39], several baby food companies provided documents on toxic heavy metal levels in baby food. It is also well-known that children’s exposure to toxic heavy metals causes permanent decreases in IQ, diminished future economic productivity, and increased antisocial behavior. The fact that babies are small, have developing organ systems, and absorb more heavy metals than adults, exacerbates their risk of exposure to heavy metals [15,40,41].

Furthermore, according to the U.S. Food and Drug Administration (FDA), “even low levels of harmful metals from individual food sources, can sometimes add up to a level of concern”. The FDA has cautioned that infants and children are at the greatest risk of harm from toxic heavy metal exposure.

Given the above information, we determined the concentrations of Pb, Cd, and Mn in different brands of CM and SBs existing on the Portuguese market and: 1. compared the concentrations of these metals between the two types of beverages; 2. compared the results obtained with permissible limit values; 3. compared our results with studies performed in other countries; and 4. alerted the authorities on the need for monitoring metal levels in these beverages as well as baby foods.

## 2. Experimental Methods

### 2.1. Reagents and Materials

Nitric acid 67%, NORMATOM^®^ for trace metal analysis, and hydrogen peroxide 30% AnalaR NORMAPUR^®^ for trace analysis were supplied by VWR BDH Chemicals. Certified reference material (CRM) TM-24.3, fortified lake water, was from the National Research Council Canada (NRCC). The magnesium nitrate matrix modifier for the graphite furnace and 2% Mg in HNO_3_ were from SCP Science (Baie-D’Urfe, QC, Canada). Stock standard solutions of lead (1 g/L), cadmium (1 g/L), and manganese (1 g/L) were supplied by SCP Science (Baie-D’Urfe, QC, Canada). All the solutions were prepared with ultrapure water (resistivity 18.2 MΩ·cm) produced with a Direct-Q 3 UV Millipore^®^ System.

Both individual stock standard solutions and working standard solutions were maintained at 4 °C. The working solutions were prepared in 5% nitric acid before each analytical run.

To avoid any possible extra metal contamination, all used materials were immersed in a freshly prepared 15% *v*/*v* HNO_3_ solution for 24 h and then rinsed thoroughly with ultrapure water and dried in a dust-free area before use.

### 2.2. Samples and Samples Preparation

In total, 28 beverages, 14 of CM and 14 of SBs, all from different brands, were purchased on the Portuguese market in Lisbon between February and May 2019. The samples were grouped according to the type of beverage, representing each sample as a different brand of beverage. The 14 CM brands were numbered from CM1 to CM14 (N = 14), and the 14 SB were numbered from SB1 to SB14 (N = 14). The beverages were thoroughly homogenized before analysis.

A digestion method described in the AOAC Official Method 999.10 [42] applies to a variety of foods; however, all these foods are solid. Therefore, we considered it valuable to develop a method applicable to liquid samples that could be used simultaneously on different organic matrices, i.e., CM and SBs. In addition, this method is less expensive and eco-friendly with the use of fewer diluted reagents; namely, nitric acid is not diluted in the mentioned AOAC Official Method, and the amount of hydrogen peroxide is double. Therefore, before metal quantification, a modification of the method proposed by Anastácio et al. (2018) [43], whose samples were also liquid (fruit juices), was used for sample digestion. Indeed, concerning the adaptation of the Anastácio et al. methodology (2018) [43], we used a smaller amount of the sample, a more diluted acid, HNO_3_, a smaller volume of H_2_O_2_, and a reduced time with the digestion vessels at room temperature. After testing different reagent concentrations and microwave digestion programs, the used method was the one that resulted in complete digestion in less time and more diluted reagents. The procedure used to digest the samples was as follows: 0.5 mL of the sample (CM or SB) was measured into a polytetrafluoroethylene (PTFE-Teflon) digestion vessel and 6 mL of HNO_3_ (2N) and 1 mL of H_2_O_2_ (30%) were added. Afterward, the samples were kept at room temperature for 3 h, and the vessels were closed. This procedure partially digested the sample before microwave radiation was applied, making the reaction less hazardous. After that time, the digestion temperature program was carried out according to the program presented in Table 1. The procedure was carried out in a Berghof microwave digestion apparatus (Speedwave Two, BERGHOF Products + Instruments GmbH, Eningen unter Achalm, Germany), allowing total digestion of the samples in a short period of time, avoiding the loss of metals by volatilization, and reducing the amount of added acid.

All the PTFE-Teflon vessels were kept closed for cooling during the night. The next day, the content of each vessel was transferred to a 15 mL volumetric flask. To ensure minimal sample losses, the vessels were washed 3 times with ultrapure water. After each step, the colorless solutions were diluted with ultrapure water until they filled the volumetric flask, and then they were transferred to tubes and kept at 4 °C until analysis.

### 2.3. Analytical Procedure

Metal quantifications were carried out in an Atomic Absorption Spectrometer (PerkinElmer AAnalyst 700, Waltham, MA, USA) using graphite furnace atomic absorption spectrophotometry (GFAAS), with deuterium background correction, single element lumina™ Hollow Cathode Lamps (HCLs) PerkinElmer, an AAnalyst 800 Autosampler, and WinLab 32 for AA software. The analyses were performed using PerkinElmer HGA pyrolytic graphite-coated tubes with an integrated platform.

The spectrophotometer wavelength and furnace temperatures are presented in Table 2. Chemical modifier Mg(NO_3_)_2_ at 2 g/L was used for Mn. For Cd and Pb analyses, the addition of different modifiers did not have an effective effect, and thus, they were not added. A background correction deuterium arc lamp was always used. The slit width was 0.7 nm, and argon was used as the purge gas.

### 2.4. Method Validation Parameters

To ensure confidence in the obtained results, several validation parameters were determined: linearity, the range of work, the detection and quantification limits, precision, accuracy, and specificity. These parameters were determined according to the ICH (International Conference on Harmonization).

### 2.5. Statistical Analysis

Data analysis was performed using IBM SPSS^®^ Statistics version 25. Lack of data normality was assessed using Kolmogorov–Smirnov tests, and, therefore, the Mann–Whitney U test was used to compare groups. *p*-values < 0.05 and a 95% confidence interval were set as the criteria for statistical significance.

## 3. Results

### 3.1. Method Validation Parameters

Under the optimized conditions described before, standard solutions of Pb, Cd, and Mn were analyzed to obtain calibration curves based on linear regression analysis of absorbance versus concentration. Calibration curves were performed with five calibration levels in the range of 5.0 to 25 µgL^−1^ for Pb, 1.0 to 5.0 µgL^−1^ for Cd, and 5.0 to 25 µgL^−1^ for Mn.

Calibration parameters (slope, intercept, coefficient of determination, residual standard deviation, and standard error) were obtained, as presented in Table 3. Differences in variances, PG-test values, and tabulated F Tests are also presented. Values of the determination coefficients (r^2^) ranged from 0.995 to 0.999 (Table 3), which demonstrates good linearity for all metals in the ranges studied.

To study the working range, the first and the last standard of each calibration curve were independently analyzed 10 times. The results as well as the relative standard deviation are presented in Table 4.

The results showed a relative standard deviation lower than 5%, except for Cd (1 µg/L).

To verify if the work range was well-adjusted, a variance homogeneity test was performed for each metal, comparing the values of the PG test with values of the Fisher–Snedecor distribution. These results are presented in Table 5, which show that the working range was well-adjusted for all the analyzed metals since the calculated PG-test value is less than the tabulated Fisher–Snedecor distribution.

LODs and LOQs were in the range of 0.16–1.41 µg/L and 0.31–4.17 µg/L, respectively (Table 6). These values allowed us to conclude that the method used is very sensitive, enabling the quantification of the metal levels in the different samples.

The intra-day precision was determined by analyzing the lowest concentration of the calibration curves for each metal ten times within a single day. The lower concentrations of the studied metals (1 µg/L for Cd, 5 µg/L for Pb, and Mn) were chosen because these concentrations are the most critical. The results, expressed as the coefficient of variation (RSD), ranged from 2.63% for Pb to 5.80% for Cd. (Table 7).

The inter-day precision was determined with the analysis, 10 times, of the first standard of the calibration curve for each metal over three consecutive days. The obtained results can be observed in Table 8.

Inter-day precision was in the range of 2.05% for Pb to 6.59% for Cd.

The data obtained with these two precision determinations, i.e., intra- and inter-day precision, highlight the low variation that can be expected when the analysis is performed on the same day or on different days.

To evaluate the accuracy of the method, a certified reference material (CRM) was used. This filtered water matrix CRM was chosen considering the unavailability of CRMs for CM or SBs and the high amount of water, more than 85%, in both samples [44,45]. The experimentally determined values are in good agreement with the certified values (Table 9) since the respective Z-score is less than 2.

The results obtained with the analysis of the certified reference material in the same conditions selected for the samples revealed that the analytical method is valid, considering they are within the range of values mentioned on the certificate.

### 3.2. Quantification of the Concentrations of Pb, Cd, and Mn in the Different Brands of Cow Milk and Soy Beverage

After optimization of the conditions for the digestion of the samples and for the quantification of the metals under study, all the samples were analyzed with GFAAS.

Table 10 and Table 11 show the average concentrations (µg/L) of Pb, Cd, and Mn in the CM and SB brands, respectively, resulting from two independent measurements.

**Figure 1:**
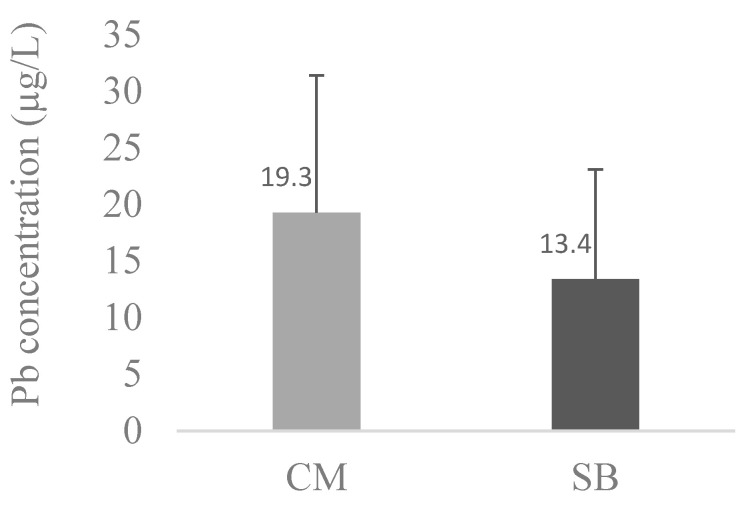
Average concentration of Pb in the different CM (N = 14) and SB (N = 14) brands. CM and SB levels of Pb were compared with Mann–Whitney tests; *p* > 0.05.

**Figure 2:**
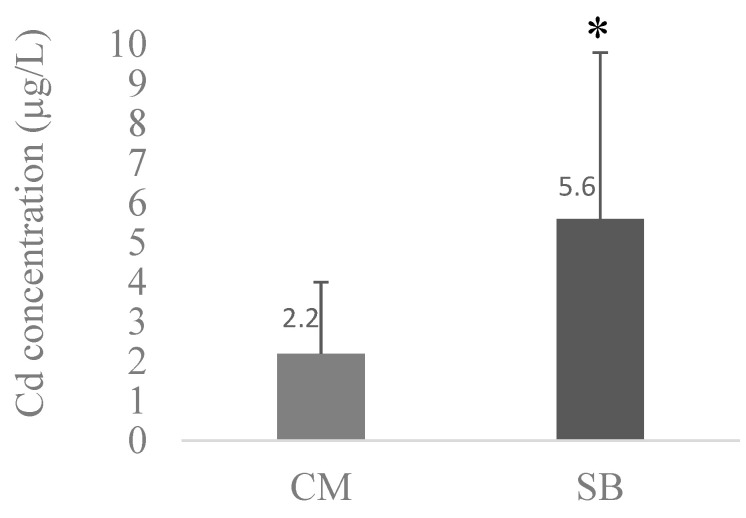
Average concentration of Cd in the different CM (N = 14) and SB (N = 14) brands. CM and SB levels of Cd were compared with Mann–Whitney tests; * means significantly different from CM (*p* < 0.05).

**Figure 3:**
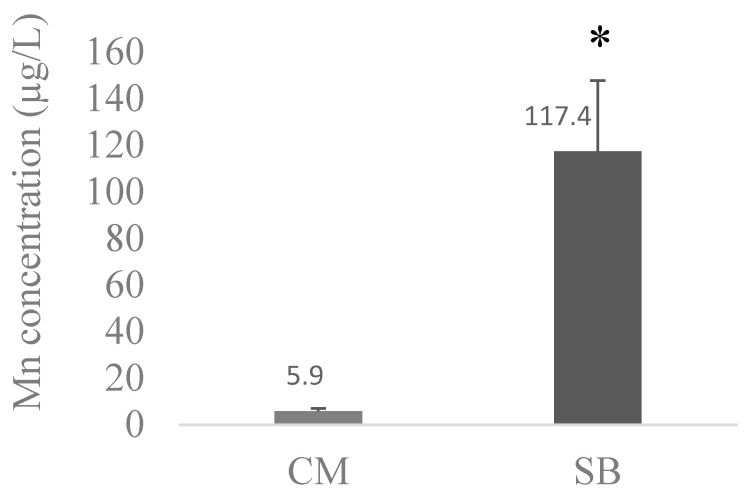
The average concentration of Mn in the different CM (N = 14) and SB (N = 14) brands. CM and SB levels of Mn were compared with Mann–Whitney tests; * means significantly different from CM (*p* < 0.05).

The average values of Pb, Cd, and Mn between CM and SBs were compared and are presented in Figure 1, Figure 2 and Figure 3, respectively.

Comparing metal levels between SBs and CM, SBs had significantly (*p* < 0.05) higher concentrations of Cd (5.6 ± 4.2 µg/L) when compared with CM (2.15 ± 1.84 µg/L); a similar trend was found for Mn (*p* < 0.05), with SBs exhibiting 117.4 ± 30.3 µg/L and CM 5.93 ± 1.21 µg/L. The inverse was observed for Pb, where the levels were higher in CM (19.3 ± 12.1 µg/L) than in SBs (13.4 ± 9.6 µg/L), although this difference was not significant (*p* > 0.05).

## 4. Discussion

Heavy metals inherent to CM are characterized by their bioaccumulation and ability to trigger cancer, mutagenicity, and developmental problems in children [2,7,24,46,47,48,49,50]. More recently, plant-based beverages like soybean beverages have been marketed as alternatives for CM [23], and they are currently one of the most consumed soybean by-products in the world [9]. Soybean plants and, consequently, SBs, contain high concentrations of contaminants, including toxic metals [9,21].

In the CM samples, the Pb concentrations (Table 10) range between 5.6 µg/L (CM4) and 39.9 µg/L (CM3), and the samples CM2 and CM3 have higher Pb concentrations of 39.8 and 39.9 µg/L, respectively. The Pb concentrations in SBs (Table 11) are between 5.4 µg/L (SB7) and 41.7 µg/L (SB1), and the latest brand has the higher concentration. We may conclude that there is great variability in the Pb levels in both CM and SB brands.

As for Pb, the average concentration of this metal in CM is 19.3 ±12.1 µg/L (N = 14) and in SB is 13.4 ± 9.7 µg/L (N = 14), which is a lower value compared with CM Pb, although not significantly different (*p* > 0.05) (Figure 1). Furthermore, according to the Commission Regulation (EU) 2023/915 on the maximum levels for certain contaminants in food and repealing Regulation (EC) No. 1881/2006 [51], the limit for Pb in milk is 0.020 mg/kg (20 µg/kg). When considering that milk density fluctuates between 1.025 and 1.035 kg/L (Parmar et al. 2006), this means that the average value of Pb in our CM brands (19.3 ± 12.1 µg/L) is close to but below this limit value.

Compared with other studies, and concerning our results for Pb in CM (19.3 ± 12.1 µg/L), we noted lower levels compared with studies such as the one by Solis et al. (2009) [52] in Mexico, where the reported values were 65 µg/L for Pb in CM, and the one by Amer et al. (2021) [7], which described a value of 45.06 µg/L in Egypt. In contrast, lower concentrations were reported in Iran by Derakhshesh and Rahimi (2012) [53], who reported an average Pb concentration of 13.45 µg/L. Additionally, Oliveira et al. (2017) reported Pb concentrations between 2.12 and 37.36 µg/L in Brazil [54], whereas Zhou et al. (2019) [24] reported Pb concentrations between 0.46 and 2.96 µg/L in China. In Brazil, Freschi et al. (2011) [26] observed Pb concentrations lower than the limit of detection (1.49 µg/L). The European legislation has not set maximum concentrations of potentially toxic elements in SB and, therefore, similar to Rubio et al.’s (2021) [9] approach, we opted to compare the determined values with other foodstuffs.

Zhao et al. (2014) [23] studied Pb (and also Cd) in soybean grains, and high concentrations of these metals were found: 340 to 2830 µg/kg for Pb and 110 to 910 µg/kg for Cd. From this, it can be inferred that SBs made from these grains will probably have high concentrations of these metals. Both studies by Rubio et al. (2021) in Spain [9] and by Turco et al. (2023) [55] in Italy, reported Pb levels of 10 µg/L in SB; meanwhile, Fioravanti et al. (2023) [56] found levels under the LOQ in all their analyzed samples except one, which exhibited a level of 7.2 µg/kg. Therefore, the values determined in our study where similar to the ones found in these other works.

The Cd concentrations in CM samples observed in our study are between 0.6 and 6.8 µg/L, where CM2 and CM3 are the brands with higher values (4.2 and 6.8 µg/L, respectively) (Table 10). In the SB samples (Table 11), the Cd concentrations are between 0.9 and 15.4 µg/L, and the SB4 and SB1 brands have the highest values (11.5 and 15.4 µg/L, respectively). The average Cd concentration in the CM samples is 2.2 ± 1.8 µg/L (N = 14), which is lower and significantly different (*p* < 0.05) from the average Cd in SB the brands (5.6 ± 4.2 µg/L) (N = 14) (Figure 2). This fact is likely related to Cd’s contamination of soy [22,23]. We compared our CM levels of Cd with the permissible level in Commission Regulation (EU) 2023/915 of 25 April 2023 on the maximum levels for certain contaminants in food and repealing [51] for infant formulas, follow-on formulas, foods for specific medical purposes intended for infants and young children, and formulas for young children placed on the market in liquid form and manufactured from cow’s milk proteins or cow’s milk protein hydrolysates, which is 5 µg/kg. Based on the results, we can conclude that average Cd levels in the different brands of CM on the Portuguese market do not exceed these limit values. Concerning the Cd average values in the SB brands in our study, we affirmed that CM2 values are higher than the proposed permissible limits for Cd in the CM formula.

For CM, average values of 4.77 µg/L were documented by Amer et al. (2021) [7], and levels of 0.05 µg/L were described in China [24]. The average Cd concentration observed in our CM samples was 2.2 ± 1.8 μg/L, which is between the values of both mentioned studies. Furthermore, the concentration of Cd in animal milk was reported to increase with increased age, confirming its bioaccumulation. For example, several authors [46,50] showed that Cd concentrations in CM from animals fed in pastures near industrial areas along highways or animals fed food contaminated with heavy metals are much higher than those that grow in clean areas.

Regarding SBs, Zhao et al.’s work (2014) [23] noted high Cd concentrations in soy grains, while both Rubio et al. (2021) and Turco et al. (2023) [9,55] reported a Cd concentration of 4 µg/L in SBs. In turn, Fioravanti et al. (2023) [56] reported values below the LOQ. The values documented in the mentioned works were similar to the values obtained in this study. Additionally, the major source of Cd pollution is fertilizers produced from phosphate ores, which may be present in pastures, explaining higher concentrations of Cd in SBs (and in CM) [57,58,59,60].

Concerning Mn concentrations in the same analyzed samples (Table 10 and Table 11), the higher concentration in the CM brands is 8.9 µg/L (CM2 brand) with an average of 5.9 ± 1.2 µg/L (N = 14), while in the SB brands, the higher concentration is 177.9 µg/L (SB5 brand), with 10 samples having values greater than 100 µg/L and an average concentration of 117.4 ± 30.4 µg/L (N = 14). In Figure 3, we noted a significant difference (*p* < 0.05) between these two types of beverage groups, with SBs having an average Mn concentration (117.4 µg/L) about 20 times higher than the Mn concentrations in the CM brands (5.9 µg/L), thus corroborating other studies. Hence, in the Peres et al. study (2016) [61], Mn in a soy-based formula was found to be 10 times higher than in a cow-based formula. According to Aschner and Erikson (2017) and Freeland-Graves et al. (2016) [37,62], plant sources have much higher manganese concentrations than animal sources. Animal foods including dairy, eggs, meats, poultry, or fish are virtually devoid of this trace element, with whole grains, vegetables, and fruits all being high in Mn [61,62].

Manganese concentrations are between 3 and 10 μg/L in breast milk, 30 to 100 μg/L in cow’s milk-based infant formulas [63], and may vary between 30 and 50 μg/L in CM formula according to Aschner and Aschner (2005) [64]. Al Sidawi et al.’s study (2021) [65] reported Mn concentrations of 36 ± 26 μg/L or 75 ± 10 μg/L in CM in Georgia, depending on the regions where the milk was derived. Knowles et al. (2006) [66] reported levels of Mn in CM between 20 and 50 μg/L. The values we obtained for the brands collected from the Portuguese market (5.9 ± 1.2 μg/L, in CM) are similar to the values found by the Freshi et al. (2011) [26] study performed in Brazil. In their study, Mn levels between 2.25 and 4.08 µg/L were described in CM.

Turco et al. (2023) [55] reported Mn levels of 1800 µg/L in SBs. High levels of Mn were reported in soy baby formulas, leading to elevated serum levels according to Mitchell et al. [67]. In fact, higher Mn values were expected in SBs [68] because grains are rich in Mn [69,70]. Aschner and Aschner (2005) [64] reported Mn concentrations in soy formula between 200 and 300 μg/L. High Mn concentrations (higher than 300 µg/L) were also found in soy-based baby formulas [71]. Mn levels in individual SB range from 2 to 17 times the mean Mn content in soy infant formulas (2.4 ± 0.7 μg/g dry wt) and 7 to 56 times that in milk infant formulas (0.70 ± 0.35 μg/g dry wt) [21]. Thus, according to Cockell et al. (2004) [21], SBs should not be fed to infants because they are nutritionally inadequate and contain Mn at levels that may present an increased risk of adverse effects if used as a sole source of nutrition.

Frisbie et al. (2019) [72] studied Mn concentrations in different products such as CM and SB in two different markets. In the USA market, 13 samples of CM were studied and were found to have Mn concentrations between 160 and 2100 µg/L, which are values that are higher than in our study (5.9 ± 1.2 µg/L). Five samples of SBs were also analyzed and were found to have Mn concentrations between 420 and 1000 µg/L, which are values that are also higher than the ones we obtained (117.4 ± 30.3 µg/L). In addition to the USA market, the same authors (Frisbie et al. 2019) [72] also studied Mn in CM commercialized in France. There, they found Mn concentrations with values between 200 and 1200 µg/L (N = 16), which are values that are higher than the ones noted in our study. No SBs were examined from the French market in their study. According to Sadrabad et al. (2018) [22], the absorption and accumulation of metals in SBs depend on the intrinsic genetic factors of each soybean plant.

The most probable explanation for the different values observed in the several studies, although on the same scale, is the fact that the samples belong to different countries (like Iran, Brazil, Mexico, and China) and thus have different geological origins, environmental factors, and genetically different cows with different nutrition states, low-quality feeds, and dietary supplements, which consequently produce different milk compositions [1,24,26,53,54]. The quality of packing may also influence the levels of the metals in milk by migration to the food [46,73].

Considering our study and other published data, although Pb and Cd in CM are in the same range and are lower than the permissible levels, they require strict control by health and environment protection institutions [74]. Furthermore, higher concentrations of Mn and Cd were observed in SBs, and these beverages are some of the most consumed soybean by-products in the world [9]. In addition, CM is highly consumed by the most vulnerable age groups: infants and the elderly [75]. As for unnecessary or toxic elements (such as Pb, Cd, and others), the latter’s presence, even in low concentrations, may lead to serious health problems in humans [65]. Furthermore, it is well-known that milk, being a food in itself, is also a very important ingredient largely used in the composition of many processed foods, including baby foods [16], thus presenting a great concern.

Given the above and based on recent information [15,39], there is great apprehension due to reports alleging high levels of toxic heavy metals in baby foods. The U.S. Subcommittee on Economic and Consumer Policy (2021) [15] requested internal documents and test results from seven of the largest manufacturers of baby food in the United States, including both the makers of organic and conventional products. As described by the Report of the Subcommittee dated 29 September 2021 [39], some baby food companies provided documents revealing a concerning lack of attention to toxic heavy metal levels in baby food and an abandonment of its previously more protective standards. In view of internal company documents and test results obtained by the Subcommittee [15], commercial baby foods are tainted with significant levels of toxic heavy metals, including Pb and Cd. As it is well-known, children’s exposure to toxic heavy metals causes several neurological problems including permanent decreases in IQ, diminished future economic productivity, and increased risk of future criminal and antisocial behavior.

In addition, we note the statement of a US Subcommittee [15], which affirms that most baby food manufacturers do not test their finished products at all and also permit dangerously high levels of toxic heavy metals. It is said that they test only individual ingredients and use those results to estimate the toxic heavy metal levels in their finished products. Lead is especially harmful to vulnerable populations, including infants, young children, pregnant women and their fetuses, and others with chronic health conditions. Thus, extrapolating these facts and taking into account the results obtained in our study, although our values are below the permissible levels (but close), as CM and SBs are used not only as simple foods, but also in large-scale processing foods, this can lead to an increase in the concentration of the mentioned metals, and their estimation based on individual ingredient testing is inaccurate and dangerous for consumers, especially children [39]. Therefore, we suggest a strict control of Pb, Cd, and Mn in CM and SBs because they are foods consumed throughout the lifetime of individuals in all age groups (enhancing children), because they are crucial ingredients of many foods including baby foods, and given the deleterious effects of these metals on child development.

## 5. Conclusions

- With respect to the method validation parameters, we are confident that the calibration parameters demonstrate good linearity for all metals in the ranges studied. The determined calibration parameters and the sensitivity of the method allowed the standard solutions and the samples to be quantified with confidence. Regarding the analytical methodology, we can confirm that the same methodology can be used for the two different matrices used in this study, CM and SBs. Having obtained optimal results with AAS, we recommend this methodology as it is low-cost when compared with Inductively Coupled Plasma (ICP). Another advantage of this methodology is the use of a small amount of sample, which consequently decreases the amount of all the necessary reagents and makes the method cheaper and more environmentally friendly.
- Regarding the results of the concentrations of the three metals observed in the different brands of CM and SBs collected on the Portuguese market, we conclude that SBs have higher and significantly different concentrations of Cd and Mn compared with CM. In addition, Pb concentrations, although higher in CM, are not significantly different between the two types of beverages.
- Regarding permissible levels, our results establish that the SB and CM average metal concentrations are below international permissible levels. Compared with other studies in multiple countries, the values of the three metals in CM and SB brands obtained on the Portuguese market have the same order of magnitude.
- As Cd and Mn concentrations observed in our samples are higher in SBs than in CM, we highlight the World Health Organization recommendation (2021) suggesting that, for children before 12 months of age, whole plain CM is preferable [76].
- Finally, but quite essential, we suggest that although the metal concentrations in CM and SBs in our study are below the permissible limit levels, health authorities must exercise strict control over Pb, Cd, and Mn concentrations in CM and SBs because these foods are ingested throughout the lifetime of individuals in all age groups, and especially early in development. In addition, it must be emphasized that CM is a crucial ingredient of many processed foods, including baby foods, and heavy metal contamination may give rise to increased accumulation of these metals in processed foods and, consequently, increased absorption and deposition in the human body.
- Although this study does not provide results that may or not suggest a potential public health concern, it certainly constitutes an alert for the need for further studies on this matter.

## Figures and Tables

**Table 1:** Microwave digestion conditions (ramp, time, and temperature). The power of equipment was kept at 90% during all steps.

Step	1	2	3	4	5
Ramp (min)	10	5	0	2	0
Time (min)	10	15	10	15	0
Temperature (°C)	170	200	200	100	75

**Table 2:** Analytical conditions (selected wavelength, dry, ashing, and atomization temperatures) for the determination of Cd, Mn, and Pb using GFAAS.

		Dry	Ashing	Atomization
Metal	Wavelenght	Temperature	Temperature	Temperature
	(nm)	(°C)	(°C)	(°C)
Cd	228.8	110	850	1650
Mn	279.5	140	1400	2200
Pb	283.3	130	1100	1600

**Table 3:** Determined linear parameters for Pb, Cd, and Mn: a—slope; b—y-intercept; r^2^—determination coefficient; S_Y/x_—residual standard deviation; Sy^2^—standard error; N—number of points on the calibration curve; DS^2^—difference in variances; PG—test value; F Test—tabulated value of the Fisher–Snedecor distribution with a level of significance α = 0.01.

	Metal		
	Pb	Cd	Mn
a	0.018	0.014	0.043
b	−0.001	0.003	−0.079
r^2^	0.999	0.998	0.995
S_y/x_	2.886	4.328	15.95
S_y_^2^	1.291	1.936	7.136
N	5	5	5
DS^2^	21.66	48.71	662.0
PG	12.99	13.02	13.00
F test *	98.50	98.50	98.50
Linearity	Confirmed	Confirmed	Confirmed

* 1; 0.99; n-3.

**Table 4:** Working range for Pb, Cd, and Mn: the first and last standards used in the calibration curves were analyzed. Determined concentrations of the studied metals are presented, as calculated according to the calibration plot of each metal. The mean values and respective relative standard deviation (RSD) expressed as % are presented, (N = 10).

Metal	Standard Solution (µg/L)	Concentration (µg/L)		
		Mean	SD	RSD (%)
Pb	5	4.156	0.109	2.632
	25	25.43	0.441	1.733
Cd	1	0.884	0.049	5.526
	5	5.651	0.119	2.112
Mn	5	5.136	0.170	3.317
	25	22.41	0.432	1.929

**Table 5:** Variance homogeneity test for Pb, Cd, and Mn: PG represents the test value, which was compared with the F test—tabulated value of Fisher–Snedecor distribution with a level of significance of α = 0.01.

	Metal		
	Pb	Cd	Mn
PG	0.108	0.153	0.071
F test	0.187	0.187	0.187
Work range	Well ajusted	Well ajusted	Well ajusted

**Table 6:** Limit of detection (LOD) and limit of quantification (LOQ) for Pb, Cd, and Mn. The calculation was achieved using two methods: the calibration curve method and by reading 10 blanks.

	Method Determination	Metal		
		Pb	Cd	Mn
LOD (µg/L)	Calibration plot	0.767	0.294	1.375
	Analysis of 10 blanks	0.163	0.465	1.411
LOQ (µg/L)	Calibration plot	2.325	0.892	4.166
	Analysis of 10 blanks	0.308	0.689	3.647

**Table 7:** Average intra-day precision values (N = 10) for Pb, Cd, and Mn. The mean was obtained for a single day, and the standard deviation (SD) and the corresponding relative standard deviation (RSD %) are presented.

	Metal (µg/L)		
	Pb	Cd	Mn
Mean	4.16	0.78	5.14
SD	0.11	0.05	0.16
RSD (%)	2.63	5.80	3.19

**Table 8:** Average inter-day precision values (N = 10) for Pb, Cd, and Mn. The mean was obtained for each day, and the mean of the three days and the corresponding relative standard deviation (RSD %) are presented.

	Day	Metal (µg/L)		
		Pb	Cd	Mn
Mean	1st	4.29	0.88	5.28
	2nd	4.16	0.78	5.14
	3rd	4.32	0.85	4.82
	All days	4.26	0.84	5.08
SD	All days	0.087	0.055	0.237
RSD (%)		2.05	6.59	4.66

**Table 9:** Analysis of Pb, Cd, and Mn in certified reference material (NRCC, TM-24.3). The table presents the certified value expressed as mean (N = 3) and standard deviation (SD), the experimentally determined values, and the Z-score.

Metal	CRM			Z-Score
	Designation	Certified Value	Determined Value	
		(mean ± SD)		
		(µg/L)		
Pb	TM-24.3	5.82 ± 0.45	6.48	1.47
Cd	TM-24.3	3.97 ± 0.37	4.22	0.68
Mn	TM-24.3	8.12 ± 1.4	7.05	1.47

**Table 10:** Quantification of Pb, Cd, and Mn in different brands of CM samples (N = 14); Samples were determined in duplicate. The mean ± standard deviation (SD) of each metal is represented for each brand and for the global sample. Each sample is compared with the limit value (LV); for Mn, there is no limit value.

Cow Milk Brand	Metal (µg/L)		
	Pb	Cd	Mn
	(LV 19.4 µg/L) *	(LV 4.9 µg/L) *	
CM1	30.3 ± 7.1	4.2 ± 0.8	7.1 ± 1.7
CM2	39.8 ± 9.5	6.8 ± 0.9	8.9 ± 2.2
CM3	39.9 ± 12.0	4.3 ± 0.7	7.2 ± 1.9
CM4	5.6 ± 1.1	0.7 ± 0.1	5.6 ± 1.0
CM5	9.7 ± 2.1	2.0 ± 0.1	6.7 ± 1.4
CM6	12.2 ± 2.5	1.4 ± 0.3	6.6 ± 0.8
CM7	25.1 ± 5.2	2.3 ± 0.5	5.4 ± 0.9
CM8	9.5 ± 1.9	0.9 ± 0.1	5.0 ± 1.2
CM9	6.7 ± 0.9	3.1 ± 0.1	5.4 ± 0.3
CM10	15.9 ± 2.3	1.2 ± 0.1	5.2 ± 1.5
CM11	15.6 ± 3.3	1.3 ± 0.2	4.7 ± 0.6
CM12	29.7 ± 5.5	0.6 ± 0.1	5.1 ± 1.7
CM13	23.7 ± 5.8	0.7 ± 0.1	4.6 ± 1.6
CM14	6.7 ± 1.4	0.6 ± 0.1	5.5 ± 1.2
Global mean	19.3	2.2	5.9
Global SD	12.1	1.8	1.2

* Considering a mean density of CM of 1.030 kg/L, the LV of Pb in CM of 20.0 µg/kg, and an LV of Cd in CM-based products of 5.0 µg/kg [44,45].

**Table 11:** Quantification of Pb, Cd, and Mn in different brands of SB samples (N = 14). Mean and standard deviation (SD). Samples were determined in duplicate. The mean ± standard deviation (SD) of each metal is represented for each brand and for the global sample. There are no established limit values for these metals for SB.

Soy Beverage Brand	Metal (µg/L)		
	Pb	Cd	Mn
SB1	41.7 ± 9.4	15.4 ± 2.8	126.6 ± 31.8
SB2	21.4 ± 3.5	5.9 ± 1.0	121.9 ± 28.5
SB3	17.3 ± 3.7	11.3 ± 2.5	106.5 ± 18.1
SB4	17.1 ± 3.8	11.5 ± 1.4	135.4 ± 26.4
SB5	8.0 ± 1.8	6.4 ± 0.3	177.9 ± 37.7
SB6	6.9 ± 1.4	3.0 ± 0.6	104.2 ± 17.6
SB7	5.4 ± 1.1	0.9 ± 0.1	177.3 ± 18.8
SB8	6.2 ± 1.0	3.9 ± 0.1	86.1 ± 12.7
SB9	18.2 ± 4.1	3.1 ± 0.6	110.4 ± 25.6
SB10	9.9 ± 2.3	4.4 ± 0.6	96.3 ± 19.2
SB11	11.9 ± 1.7	3.2 ± 0.1	72.3 ± 11.0
SB12	10.3 ± 1.1	3.3 ± 0.2	122.7 ± 23.1
SB13	7.8 ± 1.5	4.8 ± 0.4	108.6 ± 6.4
SB14	6.1 ± 1.2	1.7 ± 0.3	79.7 ± 17.3
Global mean	13.4	5.6	117.4
Global SD	9.7	4.2	30.4

## Data Availability

Data obtained in our work is presented in this manuscript.
